# A Non-cognitive Behavioral Model for Interpreting Functional Neuroimaging Studies

**DOI:** 10.3389/fnhum.2019.00028

**Published:** 2019-03-11

**Authors:** Robert G. Shulman, Douglas L. Rothman

**Affiliations:** ^1^Magnetic Resonance Research Center, Department of Radiology, Yale University School of Medicine, New Haven, CT, United States; ^2^Department of Molecular Biophysics and Biochemistry, Yale University, New Haven, CT, United States

**Keywords:** functional magnet resonance imaging, cognitive psychology, neuroenergetics, behavioral psychology, consciousness, object recognition

## Abstract

The dominant model for interpreting brain imaging experiments, which we refer to as the Standard Cognitive Model (SCM), assumes that the brain is organized in support of mental processes that control behavior. However, functional neuroimaging experiments of cognitive tasks have not shown clear anatomic segregation between mental processes originally proposed by this model. This failing has been blamed on limitations in imaging technology and non-linearity in the brain’s implementation of these processes. However, the validity of the underlying cognitive models used to describe the brain has rarely been questioned or directly tested against imaging results. We propose an alternative model of brain function, that we term the Non-cognitive Behavioral Model (NBM), which correlates observed human behavior directly with measured brain activity without making assumptions about intervening cognitive processes. Our model derives from behavioral psychology but is extended to include brain activity, in addition to behavior, as observables. A further extension is the role of neuroplasticity, as opposed to innate cognitive processes, in developing the brain’s support of cognitive behavior. We present the theoretical basis with which the SCM maps cognitive processes onto functional magnetic resonance and positron emission tomography images and compare and contrast with the NBM. We also describe how the NBM can be used experimentally to study how the brain supports behavior. Two applications are presented that support the usefulness of the NBM. In one, the NBM use of the total functional imaging signal (not just the differences between states) provides a stronger correlation of neural activity with the behavioral state of consciousness than the SCM approach in both anesthesia and coma. The second example reviews studies of facial and object recognition that provide evidence for the NBM proposal that neuroplasticity and experience play key roles in the brain’s support of recognition and other behaviors. The conclusions regarding neuroplasticity are then generalized to explain the incomplete functional segregation observed in the application of the SCM to neuroimaging.

## Introduction

The ability of imaging to measure reliable, physical chemical properties of neural activity in the brain during a person’s behavior created a revolutionary opportunity for psychology. With these new techniques, neural activity was recognized as a novel observable in the study of behavior. While measurements of animal and human behavior previously had been the most reliable observation upon which an understanding of brain function could be built, the reliability of measuring neural activity offered by brain imaging studies is now certainly as trustworthy. Functional magnetic resonance imaging (fMRI), magnetic resonance spectroscopy (MRS), and positron emission tomography (PET) measure the energy consumed by neural activity through glucose and oxygen consumption evaluated by coupled parameters of blood flow and volume. Oxidative glucose metabolism (measured by fMRI and PET) quantitatively tracks both neural electrical activity and glutamate/GABA neurotransmitter release (Rothman et al., 2011; Hyder et al., 2013a,b; Yu et al., 2017).

These and other available measurements of brain functional activity (e.g., EEG, MEG) bear upon a question often asked in psychology as to the role of an individual’s internal processes in behavior. The role of internal processes in psychology became prominent 60 years ago when behaviorism, the then popular version of psychology, was severely challenged for having ignored the inner workings of the person by having insisted that only observable behavior could be the source of scientifically valid information. Particularly influential were Noam Chomsky’s criticisms of behaviorism for having neglected internal processes during the development of language, which he claimed specifically needed contributions beyond measureable external influences and behavior (Chomsky, 1959). Chomsky (1971) proposed that psychology must include innate, internal mental processes, a position that has dominated the field ever since. Subsequently many others introduced models of cognition based on underlying mental processes. The most extreme version of this theory treats the processes, referred to as mental modules as totally isolatable both in terms of information processing and as implemented in the brain, (Cosmides, 1980; Fodor, 1983). However, although such an extreme degree of independence is rarely present in theories today at least some degree of separability of cognitive processes is almost always assumed (Gazzaniga and Mangun, 2014).

The measurement of direct brain correlates of innate internal mental processes remained elusive until the late 1980s with the introduction of PET functional imaging (Phelps et al., 1979; Fox et al., 1986) and soon after fMRI (Tank et al., 1992). The initial PET studies looked for functional segregation within the brain initially in the tradition of localizationist pioneers such as Broca, Weirnicke, etc. who, based on brain lesions, assigned different brain regions to language and other functions (for a review, see Friston, 2005 and references therein). The formal application of cognitive psychology to functional neuroimaging was introduced by Posner and Raichle (1994) and Posner and Raichle (1998) using an approach based upon the subtractive method introduced in the previous century by Donders (1969) which compared response times as a function of task complexity. The method was soon introduced into the SPM statistical methodology by Friston (1997) and Frackowiak et al. (2004; Ashburner, 2012). Although since that time there have been many different statistical and experimental approaches applied to functional imaging almost all share the assumption that the brain supports pre defined cognitive processes that depend on a degree of functional segregation. We refer to models assuming underlying cognitive processes, derived from cognitive psychology, as the Standard Cognitive Model (SCM).

Despite the continued extraordinary growth of fMRI, which has transcended the initial psychological and neurological applications to move into the social sciences (e.g., neuroeconomics) and popular culture (Satel and Lilienthal, 2013), there has been increasing concern about the disagreements between expectations of cognitive theories and experimental fMRI data (e.g., Friston et al., 1996; Shulman, 1996; McGonigle et al., 2000; Poldrack, 2006; Friston, 2009; Gonzalez-Castillo et al., 2012). In particular clear functional segregation of cognitive processes has been elusive. Most of this criticism has focused on technical issues such as imaging quality and statistical analysis, with additional work focusing on interactions between regions and non-linearity between cognitive processes and the brain’s support of them (Price and Friston, 2007; Friston and Price, 2011). In contrast there has been very little criticism of the psychological assumptions embedded in the studies (Shulman, 1996; Shulman and Rothman, 1998; Uttal, 2001; Suhler and Churchland, 2011; Shulman et al., 2014). Shulman (1996) proposed that the use of fMRI to localize brain regions to previously determined mental processes was premature and the opportunity to use functional neuroimaging to develop and test new theories was being neglected. In subsequent papers, we further elaborated this criticism using specific examples from the literature in which the expectations of clear functional segregation of cognitive processes were not being met by a modular brain (Shulman, 1996; Shulman and Rothman, 1998; van Eijsden et al., 2009). However, the goal of using functional neuroimaging to localize or find the patterns of activity that support assumed mental processes if anything has become more dominant. A recent survey of the literature from 2007 through 2011 by Tressoldi et al. (2012) found that only 11% of fMRI studies actually tested cognitive theories, the rest being used only for localization of the assumed processes. Of the 11% only a few met the criteria they set for rigorous testing of models (Tressoldi et al., 2012). Although testing of cognitive theories is not the sole value of neuroimaging studies the relatively few papers with this goal support our contention that assuming the brain has anatomical representations of cognitive concepts has rarely been questioned in imaging studies.

The question of whether the brain supports separable cognitive processes would be moot if fMRI studies showed consistent reproducible localizations that could be assigned to specific cognitive processes independent of context. However, as has been pointed out this expectation has not been met (e.g., Shulman, 1996; van Eijsden et al., 2009). The majority of approaches to address this problem have looked at modifying the statistical methods by which brain regions associated with cognitive processes are localized (Friston et al., 1996; Price and Friston, 2007) as well as broadening the criteria of functional segregation to accept a considerable amount of anatomical overlap between regions supporting different concepts. We have taken a different approach and instead have proposed that the underlying psychological assumptions regarding the cognitive structure of the brain used in designing and analyzing functional neuroimaging experiments need to be re-examined (Shulman et al., 2009, 2014, Shulman, 2013). To explore alternatives to the top down SCM approach we have proposed that neuroimaging data of brain activities should be directly correlated with behavioral observations without assuming underlying separable mental processes.

The primary goal of this paper is to explicate and formalize this approach, which we refer to as the Non-cognitive Behavioral Model (NBM) to allow its broader use in designing and interpreting functional neuroimaging studies. We start by describing assumptions that underly the application of cognitive theories to functional neuroimaging (see the section “Basic Structure of Standard Cognitive Models (SCM) and Their Application to Functional Neuroimaging”). The lack of agreement of functional neuroimaging data with the expectations of finding functionally segregated support of cognitive processes is described along with the modifications of the SCM assumptions of how the brain supports cognitive concepts to obtain better agreement with experimental data. In Section “The Non-cognitive Behavioral Model (NBM),” we describe the NBM and compare and contrast it with the SCM. Key differences include no assumptions in NBM of underlying cognitive processes, the incomplete separability of brain activity and behavior, and the importance of neuroplasticity and experience in determining patterns of brain activity. Another key difference, specific to neuroimaging, is that the total as opposed to the difference in the functional neuroimaging signal is analyzed. In Section “Application of the NBM to Studies Determining Neural Correlates of Consciousness,” we compare the NBM and SCM approaches for localizing neural correlates of consciousness from imaging data. The NBM approach of looking for correlations between the total activity of all brain regions and the measured behavior, provides stronger correlations than between brain regions proposed by cognitive theories to support consciousness and the average cortical activity.

In Section “fMRI Studies of Facial and Object Recognition,” recent studies on the fMRI responses to faces in the fusiform gyrus support the important role of experience and neuroplasticity in the development of brain responses. In Section “Application of the NBM to Study Cognitive Behaviors,” we generalize the NBM approach and point out the potential key role of neuroplasticity in explaining both similarities and differences between and within individuals performing ostensibly the same behavior. In Section “Epistemological Basis of the NBM,” we describe the epistemological basis of the NBM. We conclude by suggesting how NBM provides a useful approach for studying the brain support of cognitive and other behaviors without assuming an underlying cognitive theory.

## Basic Structure of Standard Cognitive Models (SCM) and Their Application to Functional Neuroimaging

The standard methodology used in interpreting functional neuroimaging experiments is based on the assumption, from cognitive psychology, that the brain supports cognitive and other behaviors by integrative processing of separate mental processes. The brain has regions dedicated to supporting these processes, referred to as functional segregation (Frackowiak et al., 2004). The actual behavior that takes place is the result of the functional integration of these separate cognitive processes (Friston, 2005; Price and Friston, 2007), for a comprehensive description of modern cognitive theories, see Gazzaniga and Mangun (2014). For the example of memory, there are different cognitive processes supporting long term, short term, and working memory, and within these grosser processes many sub-processes have been proposed (Baddeley and Hitch, 1974; Goldman-Rakic, 1995; Wager and Smith, 2003; Roth and Courtney, 2007; Jonides et al., 2008; Shulman, 2013, chapter 5; Gazzaniga and Mangun, 2014; Shulman et al., 2014).

The concept of the brain supporting separable cognitive processes has been criticized primarily from a philosophical perspective (e.g., Fodor, 2000; Suhler and Churchland, 2011). However, in neuroimaging it is broadly accepted with the caveat that the brain’s implementation of cognitive processes may involve considerable overlap and non-linearity (Friston et al., 1996; Friston and Price, 2011). We briefly describe below a common strategy for using the SCM in functional neuroimaging, primarily in order to emphasize how hypothesized mental processes and sub processes are integral to the experimental design and the interpretation of functional neuroimaging data. We then discuss the limitations that have been found in assigning cognitive processes to unique patterns of brain activity using neuroimaging and the approaches being taken within the SCM paradigm for addressing them. In Section “The Non-cognitive Behavioral Model (NBM),” we describe the NBM as an alternate approach for addressing structure function relationships by dropping cognitive processes as a starting assumption and instead taking a bottom up approach.

### Localization of Mental Processes by Functional Neuroimaging

**FIGURE 1:**
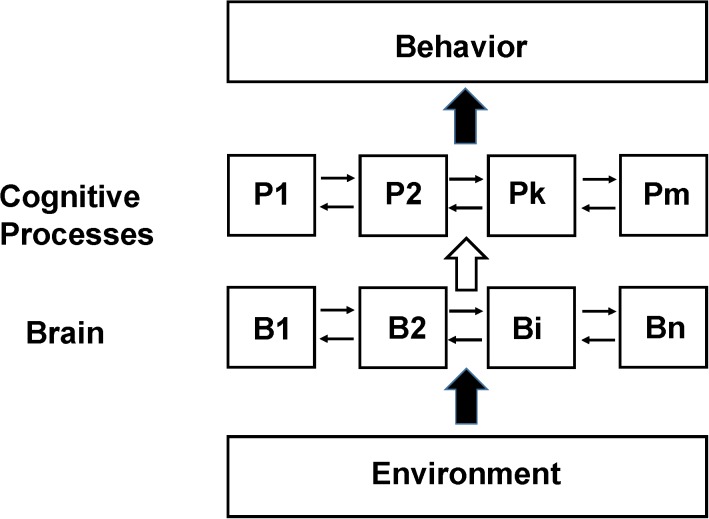
Schematic of the Standard Cognitive Model (SCM). Schematic diagram of the standard model underlying the large majority of functional neuroimaging studies especially as applied to complex behaviors involving cognition and emotion, which we refer to as the SCM. N localized regions of the brain (B1 to Bn) are organized to support m cognitive processes (P1 to Pm). The actual number and function of the theorized cognitive processes as well as the details of their interconnections depend on the specific model used. The cognitive process abstraction allows cognitive behavior to be studied at the level of information processing without reference to how specifically the brain supports it (similar to a computational algorithm not depending upon the computer hardware it is implemented on) (Baddeley and Hitch, 1974; Fodor, 1983; Pinker, 1997). In early functional neuroimaging experiments it was assumed that the brain was organized such that there was sufficient functional segregation to allow a 1 to 1 mapping between discrete brain regions and specific processes (e.g., between B1 and P1). Subsequently due to disagreements found between functional neuroimaging data the assumption of 1:1 mapping this assumption was relaxed allowing linear and non-linear interactions between different brain regions in supporting specific cognitive processes (e.g., between B1, B2, and B7 in their support of P1 and the modulation of their activity if other processes are active) (Friston et al., 1996). Experimental variables are the input to the subject being studied (referred to broadly as Environment but in many studies consisting primarily of an investigator presenting tasks or stimuli to the subject). Measurements are observable behavior and the pattern of brain neuronal activity, usually differential activity in a region relative to some other state, determined from functional neuroimaging.

A schematic diagram of how the brain is assumed to be functionally organized in the SCM is shown in Figure 1. There are three fundamental components: measured behavior at the top level, mental processes supported by brain activity at an intermediate level and regional brain neuronal activity at the lowest level. The interaction of the environment with the subject (including any sensory stimulation or psychological tasks given to them during the imaging studies) is shown as a lower level input to the brain although the actual situation is more complex including feedback from the subject’s own responses as well as any internal behavior. Brain neuronal activity is organized to support these mental processes and are assumed to have some degree of functional segregation although considerable interaction between regions may be incorporated (Friston et al., 1996). The functional integration of the outputs of the cognitive processes leads to behavior or perception.

In a typical experiment a subject in an MRI or PET scanner will perform a variety of tasks (e.g., remembering lists of words) and/or be exposed to stimuli designed to differentially activate cognitive processes. The functional neuroimaging signal (in response to an experimental input j) for each voxel is described in analysis packages as a sum of the imaging signal contributions from the region used to support the mental processes engaged to perform the task. However, the relationship between the signal, the neuronal activity underlying it, and how much of the neuronal activity is supporting a cognitive process, is usually left undefined.

In order to clarify these relationships we express the total neuronal activity (N_ij_) induced in a voxel or region i (see Figure 1) by a task j as the sum of the neuronal activity within that voxel supporting each separate mental process Xk (see also the previous description in Shulman et al., 2014).

(1) $$\mathrm{Nij}=\mathrm{boXo}+\sum_{k=1}\mathrm{bijkXk}$$

The value of Xk is nominally set to 1 (see the GLM example below). The actual value of these constants cannot be derived from a bottom up approach so that the relationship between the cognitive process and regional neuronal activity supporting it is entirely empirical. If a cognitive process k is not supported by brain activity in voxel i during task j then bijk = 0. The term boXo refers to neuronal activity assumed to not support any cognitive process. This activity is usually assumed to be equal to the neuronal activity in the voxel when no cognitive task is being performed, and is often referred to as the resting state activity. As described below quantitative imaging studies have found that the size of the term boXo is generally an order of magnitude larger than the incremental activity believed to support cognitive processes.

The relationship between the imaging signal and neuronal activity for fMRI is highly dependent on experimental methodology as well as neurophysiological couplings between blood flow, glucose oxidation, and neuronal signaling during the task (Hoge et al., 1999; Hyder and Rothman, 2012). However, in order to focus on the relationship between neuronal activity and the imaging signal we will assume that the imaging method can be corrected for vascular and metabolic response functions and calibrated such that there is a direct relationship between neuronal activity (e.g., number of spikes per second in an ensemble or number of neurotransmitter quanta released) and the signal measured and equation 1 can be used. These corrections have been performed for fMRI and PET CMRglc measurements of energy consumption^1^ (Sibson et al., 1998; Hyder et al., 2013a,b).

### Localization of Mental Processes Using Linear Models

In the original PET and fMRI functional neuroimaging studies the series of equations that can be generated from equation 2 were solved through a method sometimes referred to as cognitive subtraction. The primary assumption of cognitive subtraction was based upon the concept in cognitive psychology of pure insertion (Donders, 1969) – that separable mental processes exist and to a first order are not influenced by the activity of other mental processes. Cognitive subtraction was replaced by the use of general linear models (Friston et al., 1996; Friston, 1997) in order to provide a valid statistical framework for assessing the certainty with which the imaging results supported localization of brain activity. Subsequently there has been a move away from linear models which have been criticized (see the section “Explanations for the Lack of Clear Functional Segregation in Cognitive Neuroimaging”) primarily on the grounds that it assumes linearity in how the brain supports cognitive processes (Friston et al., 1996; Poldrack, 2006; Price and Friston, 2007). We briefly describe the general linear model below as used in functional neuroimaging below using the mathematical framework introduced above in order to clarify the neuronal basis of both the subsequent modifications within the SCM and how it differs from the NBM description.

#### General Linear Model (GLM) in Functional Neuroimaging

The concept of cognitive subtraction has largely been performed using statistical packages based upon a mathematical description called the General Linear Model (GLM) which was formally introduced into functional neuroimaging by Friston et al. (1996) and Friston (1997). It remains a major method used for the analysis of fMRI studies although as discussed below many modifications as well as alternate methods are now in use. We briefly describe its application here in order to help illustrate the differences between the underlying assumptions and experimental applications and analyses of the SCM with the NBM described in the Section “The Non-cognitive Behavioral Model (NBM).”

In a standard implementation of a GLM to functional neuroimaging the relationship between the signal in voxel i during task j (Nij) and the underlying cognitive processes (Xk) is assumed to be linear (e.g., the value of bijk in equation 1 is a constant). Furthermore, the brain activity supporting the different cognitive processes do not interact. Therefore the individual terms can be isolated through fitting for the values bijk provided that enough different tasks/stimuli (j) are performed.

**FIGURE 2:**
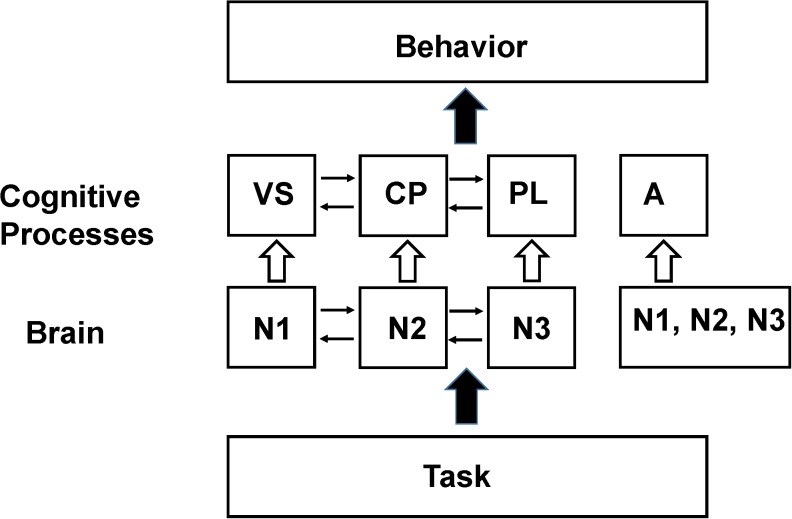
Schematic of the Baddeley and Hitch (1974) model of working memory. In their model working memory consists of a central processor (CP) that performs manipulation of data stored in a visual spatial sketch pad (VS) or a phonological loop (PL). By performing appropriate functional imaging studies the locations in the brain that support these processing modules (N1, N2, N3) can be identified provided that the assumptions in the SCM hold (see the section “Explanations for the Lack of Clear Functional Segregation in Cognitive Neuroimaging”). The resting state neuronal activity is not shown in the diagram since it has been normalized out of the imaging data. However, later functional neuroimaging studies found that these same brain regions are also recruited to support behavioral tasks hypothesized to be unrelated to working memory processes. The violation of the original assumptions about functional segregation of mental processes in the brain has led to the broadening of the assumption to allow a region to support multiple processes (Friston et al., 1996) as well as the introduction of additional processes to better fit behavioral (and sometimes imaging) data such as the concept of attention (A) which was found to activate similar regions in the frontal lobes as working memory tasks (see Shulman, 1996). In an SCM analysis the individual processing modules, N1, N2, or N3 are the experimental parameters to be varied when seeking reproducible correlations between the behavioral components and the brain regions assigned to the same processing module. The difference between these experimental parameters and those that would be used for testing the NBM model are discussed in Section “The Non-cognitive Behavioral Model (NBM).”

As an example of this procedure a simplified working memory model based on the pioneering work of Baddeley and Hitch (1974), consisting of a phonological loop (PL), visual sketch pad (VS), and central processor (CP) is shown in Figure 2. If the goal of the experiment is to locate where these processing modules the system is modeled, using a procedure referred to as the design matrix as consisting of three cognitive processes XCP, XPL, XVS. The imaging signal time course is then measured during three (or more) tasks, with the focus being on the time intervals where the contribution from each process is approximately constant. As an example the first imaging measurement is made during an auditory task involving the PL, the second during a task that activates the visual spatial sketch pad but not using the PL and the third during a task that does not activate the visual scratch pad. The signal measured during each task is then modeled with the following series of linear series of equations (equations 2–4) along with an error term ej.

(2) $$\mathrm{Task}\;1\;\mathrm{Ni1}=b0X_{o}+\mathrm{bi}11X_{\mathrm{CP}}+\mathrm{bi}12X_{\mathrm{PL}}+\mathrm{bi}13X_{\mathrm{VS}}+e1$$

(3) $$\mathrm{Task}\;2\;\mathrm{Ni1}={\mathrm{boX}}_{o}+\mathrm{bi}21X_{\mathrm{CP}}+0+\mathrm{bi}23X_{\mathrm{VS}}+e2$$

(4) $$\mathrm{Task}\;3\;\mathrm{Ni1}={\mathrm{boX}}_{o}+\mathrm{bi}31X_{\mathrm{CP}}+\mathrm{bi}32X_{\mathrm{PL}}+0+e3$$

Since the neuronal activity supporting a process is assumed to be constant the series of equations above can be solved using standard statistical matrix methods (Friston, 1997) for the constants bijk allowing the regional neuronal support of each process to be localized along with an estimate of the uncertainty in the assignment.

The determination of the coefficients is further simplified by the assumption that only the change measured in the imaging signal (and underlying neuronal activity) during a task or stimulus is relevant for supporting cognitive processes (Morcum and Fletcher, 2007). There are many methods for removing this signal, but the most commonly used equate it to the resting state (or baseline) signal when the subject receives no stimulation or performs tasks (see the section “Explanations for the Lack of Clear Functional Segregation in Cognitive Neuroimaging”), which is equivalent to removing the term boXo in equation 1.

### Explanations for the Lack of Clear Functional Segregation in Cognitive Neuroimaging

Since the initial cognitive neuroimaging studies of Posner and Raichle (1994) and Posner and Raichle (1998) there has been a general expectation that functional segregation of mental processes would be found in the brain. However, there have been a large number of reports in which reproducibility and degree of functional segregation within and between subjects for the same task has not been high (Friston et al., 1996; McGonigle et al., 2000; Otzenberger et al., 2005) particularly for cognitive but also for sensory tasks. Despite this problem studies that find different spatial responses assigned to the same function or similar spatial patterns of activation assigned to the same function have rarely led to questioning of the fundamental cognitive concepts being localized in the brain (for specific examples, see references in Shulman, 1996, 2013; Shulman et al., 2007; van Eijsden et al., 2009). Instead they have been mainly attributed to limitations in the imaging methods and incomplete functional segregation, and linearity, in how the brain implements cognitive processes (Friston et al., 1996; McGonigle et al., 2000; Gonzalez-Castillo et al., 2012). We briefly describe some of the main concerns in this section and how they have been addressed by others in the field. In Section “The Non-cognitive Behavioral Model (NBM),” we describe our alternative proposal that the assumption that the brain supports separable mental processes that can be described in terms of information processing as in cognitive psychology needs to be examined critically.

#### Influence of Resting State Neuronal Activity on the Functional Neuroimaging

During the initial decade of PET and fMRI functional neuroimaging it was widely believed that despite the brain being energetically very expensive (approximately 20% of the body’s oxygen consumption at rest) neuronal signaling activity was not energetically costly. This conclusion was based on several lines of evidence including measurements suggesting low energy costs of action potentials extrapolated to the human brain (Creutzfeldt, 1975), findings from PET of a very small energetic cost for cortical activation due to preferential use of glycolysis (Fox et al., 1986), as well as the concept of a large metabolic pool and small neurotransmitter/functional metabolic pool in neurons. As expressed in a review from that period (Raichle, 1998).

“These results suggested that the additional metabolic requirements associated with the increased neuronal activity might be supplied largely through glycolysis alone.”

Due to the low energy yield of glycolysis it was inferred that only a few percent of the energy supporting the brain was devoted to supporting neuronal signaling (see Hyder et al., 2013a for more recent calculations of the energy derived from glycolysis versus glucose oxidation). Therefore the large resting state imaging signals, whether CBF, CMRO_2_, or CMRglc (which except under intense sensory activation is tightly coupled to CMRO_2_), were not considered to reflect neuronal activity since they were primarily considered to be supporting the brain’s non-signaling activities.

The concept of the majority of the brains energy not directly supporting function was challenged in 1998 with the finding, using ^13^C MRS, that approximately 80% of cortical neuronal energy in the resting state was directly supporting neuronal signaling, as measured quantitatively with glutamate/glutamine cycling (Sibson et al., 1998). Subsequently this result has been replicated repeatedly and extended to GABAergic signaling and glial metabolism (Yu et al., 2017) in animals and humans as well as to independent electrical measurements of signaling (Hyder and Rothman, 2010; Hyder et al., 2011, 2013a). In addition multiple studies have shown that it is consistent with measurements of signaling and energetics at the cellular level when scaled up to whole cortex (Atwell and Laughlin, 2001; Yu et al., 2017). In 2001 Raichle et al. (2001) showed that PET measurements of the oxygen extraction fraction and fMRI measurements of deactivations were consistent with a high resting state level of neuronal activity relative to the fluctuations during tasks. In addition to providing localized signals during a task it was soon proposed that these changes were coordinated during the resting state by networks specialized for specific functions such as the default mode (Gusnard and Raichle, 2001). Similarly, Stark and Squire (2001) proposed high baseline activity as the explanation for paradoxical results found in neuroimaging studies of the mesial temporal lobe. In a recent review of imaging studies of brain function Raichle (2015) has proposed that the efficient brain use of the total energy supports signaling which clears up the previous questions on whether the presence of a high baseline signaling was regional (Raichle, 2010; Hyder et al., 2013a).

The high resting state neuronal activity (boXo in equation 1) (and associated neuroimaging signal would still not impact task based fMRI if the neuronal activity induced by a task or stimulus could be treated as being independent, as per the assumptions of linear models (Morcum and Fletcher, 2007). However, initially in animal models (Hyder et al., 2002; Zhu et al., 2009) and more recently in humans it has been shown that the increment or decrement measured during a task or stimulus is strongly dependent on the magnitude of the baseline neuronal activity (Uludag et al., 2004; Pasley et al., 2007; Hyder and Rothman, 2011). A similar dependence of the magnitude and pattern of fMRI fluctuations during the resting state upon the baseline neuronal activity measured by PET has recently been reported (Riedl et al., 2014; Aiello et al., 2015; Marchitelli et al., 2018).

Although there have been attempts to incorporate resting state activity into task/stimulus based functional neuroimaging there is no generally accepted procedure and the large majority of functional neuroimaging studies assume independence and either regress the baseline out (see Mortensen et al., 2018) or do not measure it as in fMRI. We note here that the global signal normalized out in resting state and sometimes task fMRI (Smith et al., 2013) is not a measure of the average resting state activity but rather correlated fluctuations in the low frequency fMRI difference signal across the entire cerebral cortex.

#### Non-linearity in the Imaging Response, Regional Interactions, and Networks

A key assumption in linear approaches is that the neuronal activity change induced within a voxel by a mental process (and the underlying signaling) is independent of the neuronal activity within the same voxel (and other voxels) supporting other mental processes. A range of studies have shown that this assumption can be violated, for example in studies in which there are competing processes (Kastner et al., 1999; Reynolds and Desimone, 2003; Friston, 2009). Friston et al. (1996) have proposed that non-linear violations could be analyzed using a factorial approach in which the signal is described as the sum of regionally specific activations and regionally specific interactions between component mental processes. While this approach can better fit experimental data the assumptions of the validity of the cognitive models being used were not questioned, but rather how the brain supports those models:

“The point being made here is that although a cognitive science model, describing the functions, may include serial and additive elements the implementation of those functions is not. Consequently the structure of the cognitive components (functional model) and the brain’s physiological implementation are not isomorphic and the mapping of one onto the other is problematic” (Friston et al., 1996).

More recently Price and Friston (2007) proposed a methodology by which neuroimaging could be used to help better define cognitive models using an approach they refer to as functional ontology, taking advantage of the interaction terms between neuronal activity in different brain regions. This and related work has been critiqued by Klein (2012) who argued that due to the many mappings between the same regions of the brain to different cognitive processes there is a need to include context dependence in the approach. While we feel these and related approaches to test and distinguish cognitive theories using neuroimaging data should be commended they differ from the NBM in that they largely focus on distinguishing cognitive theories rather than taking a complete bottom up approach in which theories of brain function are developed with functional neuroimaging and behavioral measurements, along with relevant neuroanatomical measurements, as the starting point.

Following from the work showing regional interactions network mapping has become an important area in functional neuroimaging both during tasks and at rest (Biswal et al., 1995; Hampson et al., 2006; Smith et al., 2013). However, in the majority of cases the networks identified are still assigned to supporting mental processes. Even for resting state fMRI the networks identified are usually related to a cognitive process such as the assignment of the default mode network (DMN) to the concept of general awareness/consciousness In Section “Application of the NBM to Studies Determining Neural Correlates of Consciousness,” we describe studies in which the assignment of the DMN to a consciousness module was found to not correlate with the level of consciousness in coma patients as accurately as the NBM proposal, based on analysis of the total PET imaging signal during anesthesia, that total cortical activity was the best neuronal correlate of consciousness.

## The Non-Cognitive Behavioral Model (NBM)

The lack of a clear correspondence between the mental processes in models from cognitive science and anatomical localization of these processes by functional neuroimaging have led us to take a different approach, the NBM, in which cognitive models are not assumed from cognitive psychology but instead are empirically derived from imaging and other direct measurements of brain activity (Shulman et al., 2009, 2014; Shulman, 2013). In this section, we formalize this approach and contrast it with the SCM assumptions and methodology as applied to imaging.

### Structure of the NBM

The NBM was developed from consideration of experimental results (Shulman et al., 2014) and from philosophical considerations on the psychological findings of Behaviorism (Shulman, 2013). We list the major assumptions and components of the model below.

#### Behaviorism as a Basis for NBM, the Absence of Assumed Mental Processes

Behaviorism is a psychology which proposes that behavior is a consequence or the effect of conditioning. Classical behaviorism was criticized for neglecting the state of the brain which is continuously engaging in internal behavior (for example the Freudian unconscious and in the cognitive psychology view the mental processes that compose SCM theories). We propose that due to functional neuroimaging it is possible to directly measure the internal activities of the brain, and therefore there is no need to introduce theoretical, abstract mental processes as an intermediate between inputs and behaviors. The role of experiment in NBM is to seek empirical connections between brain activity patterns developed by neuroplasticity in response to reproducible behavior or training as opposed to testing for brain regions that support predefined mental processes that act as an intermediate stage between brain activity and behavior.

This avoidance of mental concepts may be difficult to accept because these generalizations are of considerable value in daily life (e.g., concepts such as long term memory, attraction, intention, etc.) where they are firmly accepted in what is sometimes called “Folk Psychology.” The effort to define what is meant by them and where they are supported in the brain has been a principle goal of neuroscientific and philosophical enquiry. However, in our opinion they have stood in the way of the development of novel data driven approaches to understanding how the brain supports behavior.

#### Neuroimaging Can Measure Patterns of Brain Activities Supporting Behavior

This is an inference from the many neuroimaging results that have shown that different behaviors are supported by different patterns of brain activity. However, it is an open question as to how reproducible these patterns are and how uniquely they map to behaviors. To the extent they are reproducible and generalizable across different behaviors it may be possible for experiments to identify the necessary patterns of activity needed, particularly for behaviors that have been reinforced by repetition (see the sections “fMRI Studies of Facial and Object Recognition” and “Application of the NBM to Study Cognitive Behaviors”).

#### Measured Behavior Is Defined Operationally as Opposed to Being Based on Conceptual Generalizations

In SCM studies, the behavioral tasks studied are usually classified using concepts derived from cognitive psychology. In the NBM model, behavior is described only in terms of the actions performed. For example an NBM study of memory would expose the subject to all types of tasks that involve retrieving information. By contrast, in an SCM study the memory task would be classified according to differences in the cognitive processes they are hypothesized to contain such as working memory (and its sub components), long term memory and short term memory. This difference in parameters to be tested experimentally is the main operational difference between NBM and cognitive based models like SCM. Similarly, as described in Section “Application of the NBM to Studies Determining Neural Correlates of Consciousness,” when NBM approaches were used to look for brain behavioral correlates of consciousness the state of consciousness was defined by how the subject responded to a standard list of simple questions about behavior from an experienced anesthesiologist (Shulman et al., 2009, 2014; Stender et al., 2015, 2016). This operational definition contrasts with the approach in most SCM neuroimaging studies of consciousness in which consciousness is defined in terms of cognitive concepts such as self awareness.

#### For Behaviors to Be Considered the Same or Similar They Require That the Associated Patterns of Neuronal Activity Be Similar

**FIGURE 3:**
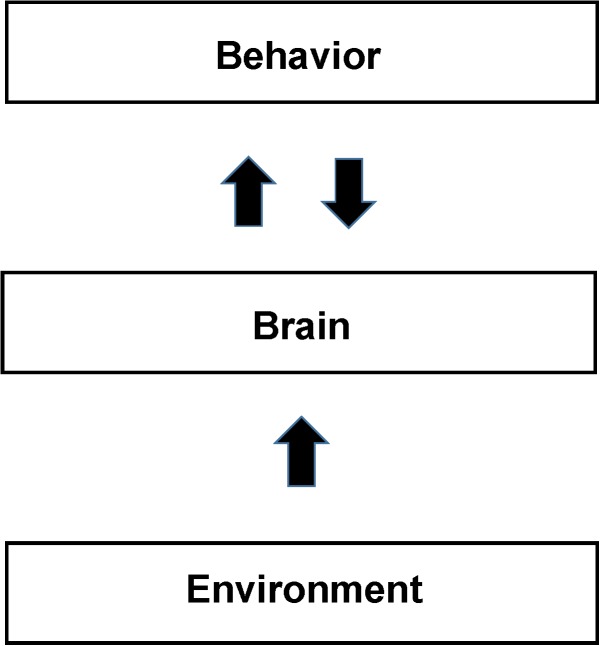
Schematic of the Non-cognitive Behavioral Model (NBM). The figure shows a schematic diagram of the NBM. It differs from the SCM primarily by not assuming that the brain is organized to support localized cognitive and other separable mental processes. Brain support of behavior is derived from direct analysis of brain total activity maps from neuroimaging. The back and forth arrow between behavior and brain reflects their interdependence in addition to their separate causal factors. Identifications of both brain activity and behavior depend on their reproducibility and the precision of measurements. Behavior and brain activity are separated by measurements as opposed to the SCM view that behavior, arising from the mind, forms the relationship between mental and behavioral states. Sections “fMRI Studies of Facial and Object Recognition” and “Application of the NBM to Study Cognitive Behaviors” present examples that use behavior and brain measurements to understand whether apparently similar behavioral states are actually similar (as opposed to being so assumed from brain models). Neuroplasticity, in response to environmental experience, including training, develops readily available neural support mechanisms for individual behaviors. Due to the large variations in the experiences of individuals (see the section “Application of the NBM to Study Cognitive Behaviors”) we expect significant intersubject differences in neural activity (and the resultant functional neuroimages), in the absence of reproducible behavior.

In the NBM behaviors are considered the same or similar only if both the measured behavior and the measured patterns of brain activity are the same or similar. For example if the brain pattern of activity differs between subjects performing the same task then the behavior is different even if the behavioral measurements are the same. In Section “Application of the NBM to Study Cognitive Behaviors,” we argue that the poor reproducibility of studies trying to pinpoint the locations in the brain that support cognitive and other complex behaviors is due to the tasks being performed differently between subjects. The difference is due to the subject’s different life experiences, and also due to feedback between behavior and brain during the study. In other words, the behavior itself is continuously modifying the patterns of brain activity supporting it due to neurofeedback. In SCM the behavior is usually described as in Figure 1 as deriving directly from brain activity via functionally segregated regions supporting intermediate mental processes. In contrast in the NBM there is a continuous interdependence of brain activity and behavior as expressed by the back-and-forth arrows in Figure 3.

#### The Definition of Behavior Includes Internal Processes and Perception

Although not directly accessible by external behavioral measurements the NBM definition of behavior, including intrinsically internal processes such as silent reading, imagining, and object recognition, are considered as behaviors in the NBM. This inclusion differs from traditional behavioralism but is justified by imaging providing the ability to measure internal behavior.

#### Context and Experience Plays a Key Role in How the Brain Supports Behavior Through Neuroplasticity and Other Low Level Mechanisms

In the SCM the components of high level cognitive processes are assumed to be generally present in all individuals, which justifies image and behavioral measurement averaging across individuals. This approach has been criticized for not taking context and individual experience into account (Shulman, 1996; Poldrack, 2006; Klein, 2012). The brain support of behavior depends upon context and individual experience acting through neuronal learning mechanisms. These mechanisms are shared between individuals but differences in their life experiences and specific task related training (e.g., object recognition as discussed in section “fMRI Studies of Facial and Object Recognition”) may cause substantial differences in how the brain supports the behavior and even in the nature of the behavior itself to the subject.

### Description of the Neuroimaging Signal in the NBM Formalism

In the NBM the neuronal activity in voxel i during behavior j (Nij) cannot be decomposed into separate linear contributions as in equation 1. If it is possible to describe the brain functionally based on the postulates of the NBM the signal in a voxel i during task j is described by a generalized function (eq. 5):

(5) Nij=Fi(T+t)

Note that ‘rest’ is also considered a behavior (or state) so there is no separation between task and resting state imaging signals. The time variable T refers to the life history prior to the application of the task j in the study. The experiences acquired during the subject’s life history influence the brain’s pattern of neuronal activity supporting a behavior based upon neuroplasticity. In addition it determines the state of the brain at the time of the study (an extreme example of a state difference is a subject who is asleep in the scanner during the task. However, it is likely that much smaller state differences can significantly influence the brain’s response).

Although, we have expressed the response here in terms of a function to clarify the differences with the SCM we do not know whether such a function actually can be found or even exists. Ultimately only experimentation and insights into brain organization and function at lower levels of description than the psychological level applied to most neuroimaging studies may be able to answer this question.

### Experimental Application of the NBM

The NBM approach is in some ways conceptually similar to early electrical mapping and neuroimaging studies of Hubel (1988) and Zeki (1993) of the brain response to sensory stimulation. The identification of the functions supported by specific brain sub regions (e.g., columns in the primary visual cortex) was determined through trial and error in the exposure to a wide range of stimuli. While these studies identified microscopic level modularity (e.g., specific columns to edge detection, rotations, color detection, etc.) the functions of these regions were not assumed in advance, but were identified by experiment.

To apply this approach to cognitive neuroimaging requires that a range of behaviors be performed and the patterns of brain activity measured. We describe the result functionally in equation 6 to facilitate comparison with the SCM.

(6) Nij=Fij(T+t) +e(t)

Behaviors (including internal behaviors such as perceptions) are not pre classified but rather tested for similarity based upon the consistency between the patterns of brain activity they induce. As described in Section “Application of the NBM to Study Cognitive Behaviors” this approach can potentially be used to determine which behaviors are related and how closely (through similarly of patterns of activity) as well as which regions are most directly involved in supporting a behavior The ability to statistically compare images without an underlying cognitive model does not present a novel challenge for imaging analysis. A wide range of methods already exist for testing pattern similarity in images (e.g., Kriegeskorte et al., 2008; Huth et al., 2016; Yourganov et al., 2014).

In order to illustrate more concretely the difference between the SCM approach we describe the application of the NBM to study working memory in comparison to that described in the Section “Basic Structure of Standard Cognitive Models (SCM) and Their Application to Functional Neuroimaging” and Figure 2 as applied to the working memory model of Baddeley and Hitch (1974). The fundamental difference is that no cognitive process or processes for working memory would be assumed and used to design the memory tasks presented to the subject. Instead the subject would be presented different tasks in which memory would be operationally defined as involving performing recall or recognition of previously presented information (e.g., a list of words) in which parameters such as modality, time between presentation, task performance, and manipulation of presented information would be varied. The spatial and temporal pattern of the total neuronal activity (at a voxel level) would be measured for each memory task as well as the performance (behavior) of the subject. No normalization to take out resting state neuronal activity would be performed as in the SCM approach (the boXo term in eq. 1).

The results from the imaging of different memory tasks within a subject would then be compared using pattern analysis to assess the degree of similarity in the brain’s response. As described in Section “Application of the NBM to Study Cognitive Behaviors” it may be possible to form classifications based upon the brain activity and behavior or tasks that are more or less similar. Based on these classifications, and assuming that results between subjects are sufficiently similar despite differences in their life history, it may be possible through further study to determine mechanisms (particularly through integration of results from different levels of study).

## Application of the NBM to Studies Determining Neural Correlates of Consciousness

There is an extensive history of studies attempting to identify neuronal correlates of consciousness (Kulli and Koch, 1991). As applied to neuroimaging these studies largely have focused on identifying the location of brain region(s) that support the mental processes responsible for the conscious state. This localization is based on the differential activity between these regions and the average activity of the total cortex, as expressed in terms of the PET CMRglc signal. In 2009, we analyzed previous experimental results from PET CMRglc studies of anesthesia using an early form of our NBM model (Shulman et al., 2009). We expand our analysis here using the NBM as described in this paper and apply it to new results that directly tested our conclusions.

The data initially analyzed were PET CMRglc images of human subjects at different levels of anesthesia and behavior. There have been multiple attempts, discussed below, to come up with an SCM based identification of specific brain regions, previously assigned to behavioral concepts like awareness, that support the state of consciousness. These studies identified the role in consciousness contributed by these regions based on their showing a greater drop in the imaging signal during anesthesia than the average of the entire cerebral cortex. However, no consensus had been reached in these studies as to the anatomical localization of these cognitive components assigned to consciousness. By contrast an NBM analysis (Shulman et al., 2009) concluded that the best correlate with the behavior of a person in the state of consciousness was the total global cortical neuronal activity (as assessed from PET CMRglc). We describe below the differences between the SCM and NBM analysis to illustrate how the differences between the models adopted *ab initio* can lead to fundamentally different conclusions of how the brain supports behavior. We then describe a recent study of the clinically defined minimally conscious state (MCS) that directly compared the SCM and NBM predictions.

Although, we recognize that the studies of consciousness (often referred to as looking for correlates of consciousness) analyzed here do not follow the standard SCM experimental procedure in fMRI of within subject design they constitute a substantial body of research using both metabolic based imaging (fMRI, PET) and EEG/MEG based imaging. Furthermore they provide a clear test of the NBM proposal that the total imaging signal contains critical information on brain function and cannot be normalized away based on SCM assumptions regarding the separability of brain activity [see the section “Basic Structure of Standard Cognitive Models (SCM) and Their Application to Functional Neuroimaging”].

### Description of Anesthesia Studies

Several laboratories (Alkire et al., 1995; Alkire, 2008; Katoh et al., 2000; Schlunzen et al., 2010) had performed PET neuroimaging studies on unconscious humans under surgical levels of anesthesia and in coma (Laureys et al., 2002, 2004) because of its clinical importance. In the anesthesia studies, subjects were studied both in the awake state and at various levels of anesthesia that reduced the average cortical (and sub cortical) energy consumption down to ∼50% of basal values, the level used for surgery. At different levels of anesthesia (and associated reduced CMRglc) subjects were assessed to be in a decreased state of consciousness based on their responses to questions and physical stimuli that are frequently used by anesthesiologists in clinical practice. Unconscious state cortical gray matter CMRglc was relatively uniform. Subsequent quantitative analysis has shown that the variations from the mean are no more than 10% (Hyder et al., 2013a).

### Comparison of NBM and SCM Interpretations of the Anesthesia Results

#### Definitions of Consciousness

In the studies, where an SCM analysis was performed the behavior was defined in psychological terms in which consciousness was assumed to be a mental process that supported concepts such as self awareness. In contrast, in the NBM analysis the state of consciousness was identified by its behavior which was the ability of the subjects to respond to questions or physical stimuli from the anesthesiologist well enough to be classified as in the state of consciousness by standard metrics in the field. No attempt was made to relate the ability to respond to underlying cognitive descriptions of consciousness. The differences between the SCM conception of consciousness as a mental process conducted by functionally segregated brain regions and the NBM definition of it as a behavioral state of the subject altered the interpretation of the imaging results as described below.

#### Assignment of the Location of Consciousness Using the SCM and NBM Paradigms

Based on the SCM assumptions mental processes supporting consciousness were localized by looking for the regions that in the anesthetized state had the lowest level of FDG uptake relative to the average FDG uptake across the entire cortex. The large drop in global cerebral activity in all regions was assumed to not impact consciousness (and therefore was normalized away). This approach led to the brain support of the consciousness process being assigned, in different studies, to the thalamus, precuneus, inferior frontal lobe, and more recently the DMN (Alkire et al., 1995; Katoh et al., 2000; Laureys et al., 2004; Alkire, 2008). The variation in regional assignment is not surprising given that the regional fluctuations relative to the normalized global average imaging signal being at most 10% which is on the order of uncertainties in the imaging signal (Shulman et al., 2009; Hyder et al., 2013a).

In contrast, the NBM interpretation took into account the entire imaging signal since there was no *a priori* reason to distinguish the roles of different neuronal activities (i.e., total neuronal activity versus regional variations in activity). It was concluded (Shulman et al., 2009) that the averaged global drop in cerebral neuronal activity, as inferred from CMRglc PET measures of total energetics, was the best correlate of the state of consciousness. This drop was much larger than differences between the activity of different regions in either the conscious or unconscious states.

### Statistical Comparison of SCM and NBM Predictions for Patients in the Minimally Conscious State and Vegetative State

The prediction from the NBM analysis that the global brain activity (as reflected in functional neuroimaging measurements of total glucose or oxygen consumption), would reflect the state of consciousness was recently tested in PET studies in a cohort of 41 patients in either a MCS or vegetative state (VS) as defined by standard neurophysiological measurements of Disorders of Consciousness (DOC) (Stender et al., 2015). Coma patients have long presented a challenge for predicting which patients will recover from the VS to reach consciousness and also whether the degree of consciousness of MCS patients can be enhanced.

In their study, Stender et al. (2015) found that the global CMRglc averaged 42% of control in the VS and 55% in the MCS. Regression analysis showed very little difference between values in several areas previously proposed to support consciousness processes, the brain stem, thalamus, precuneus, and frontal parietal cortex, and the global cortical activity, which explains the poor reproducibility of previous studies using the SCM approach of looking for regions of maximum difference. Overall they concluded that total activity in the prefrontal lobe and the entire cortex were able to accurately distinguish states of consciousness. In a subsequent validation study that included 131 DOC patients (Stender et al., 2016). They reproduced these findings and established that 42% of normal cortical CMRglc is the minimal energetic requirement for conscious awareness.

### Conclusions and Extension to the Study of Different States of Consciousness

In neuroimaging studies of anesthesia, and independently validated in coma, the identification of the total CMRglc signal (primarily reflecting neuronal signaling) by the NBM as the best correlate of consciousness was tested and confirmed in two studies of coma patients in different states of consciousness. The Stender studies found a somewhat better correlation with total cortical activity of the frontal parietal cortex, but the total CMRglc signal for both the global and frontal parietal cortex correlated better than differences in activity between regions previously assigned to selectively supporting consciousness.

A potential criticism of the NBM approach to interpreting and designing functional neuroimaging studies of consciousness is that it does not distinguish between different levels of consciousness and SCM derived properties, such as self-awareness, that are usually included in the definition of consciousness when physical correlates are looked for Crick and Koch (1990), Kulli and Koch (1991), Koch (2004). From an experimental standpoint, an NBM based study could be done by looking for correlations between images of brain activity (using a variety of imaging methods) and the level of response to any of a number of behavioral measures. Plots of the percent correctness of answering typical verbal questions at different levels of anesthesia (Katoh et al., 2000) have shown a non-linear but monotonic correlation of anesthesia with the variables responsible for the state of consciousness.

Although the global brain activity is the key parameter correlating with being in the state of conscious the NBM does not rule out that patterns of brain activity may vary at different levels of conscious activity. In their 2015 paper, Stendor et al. found that the standard deviation in the total cortical activity increased as a percentage of the global average at higher states of consciousness. This finding is consistent with MCS patients often having the full range of sensory and cognitive abilities (e.g., language) although at a lower functioning level supporting a key role for global cortical activity in specific behaviors (which in the SCM is treated as independent of cortical activity).

In addition to resolving the questions evoked by the term consciousness the NBM framing of research questions in terms of observable behaviors and the identification of an experimental parameter, the total global energy, which, quantitatively measured, opens the door to novel experimental studies. By allowing experimental results to define the parameters of interest instead of insisting on fitting a cognitive model the NBM promises to continue to provide novel insights into consciousness. The important novelty of our approach is that we identify a brain property when the person is in the behavioral state of consciousness that changes when he or she leaves that state. This along with analyzing the total brain activity within brain regions has allowed a stronger correlate of consciousness to be identified than the SCM interpretation which assumes that a localized modular response varies with conscious behavior. Future studies of the relationships between the global and regional levels of brain activity at different levels of consciousness (Kotoh) may reveal further insight into their interplay in supporting specific conscious behaviors.

## fMRI Studies of Facial and Object Recognition

In this section, we assess whether the SCM assignment of the fusiform gyrus (FG) region as supporting a facial recognition process or module is a better explanation than the training and neuroplasticity proposed in the NBM. Although these studies were not performed using the NBM paradigm we believe they provide important support for the importance of neuroplasticity and personal experience in developing the brain’s support of behavior.

The fusiform gyrus is a brain region intermediate between visual and cognitive processing that has shown good image reproducibility. Early functional imaging studies had identified a facial recognition region in the FG by the difference between the neuronal signal from faces right-side up and up-side down (Allison et al., 1994; Puce et al., 1996). The differences in the FG between up and down orientations of human faces gave a reproducible fMRI difference signal leading to this locale being defined as the fusiform face area (FFA) region. The ease of finding this response to faces led to the assignment of this region of the FG as “the FFA: a module in human extrastriate cortex specialized for face perception” (Kanwisher et al., 1997). However, differing from this interpretation, other studies showed that the FFA responses that experts have learned to familiar objects (e.g., of bird watchers to birds) supported a similar reproducible response to familiar “non-face objects.” The similar responses in the FFA of untrained persons to faces and of experts to familiar objects have been interpreted either as the activations of innate modules, consistent with the SCM (Kanwisher et al., 1997) or as the effects of expertise developed from training, consistent with the NBM, in which the FFA is part of a network tuned by experience to individuate visually similar objects. Experiments were performed that distinguish these two interpretations which we review here.

Based on the convention of researchers in the field (e.g., Kanwisher et al., 1997) we refer to the mental process of facial recognition in this section as a module. The term module based on historic usage (see Fodor, 1983, 2000) refers to a mental process that is almost completely isolated from other processes (other than inputs and outputs) and is anticipated to have a similar degree of isolation in its implementation by the brain. Supporting this assumption for the fusiform gyrus, which is part of the visual processing stream, a high (although not complete) degree of modularity has been found in regions of the visual cortex particularly V1.

### fMRI Studies of the Effect of Training on the FFA Response

To clarify the experimental distinction between the two brain models, studies aimed to distinguish whether the enhanced neural response in the FG was due to an innate module for facial recognition or due to the expertise created by training. Among the early studies addressing this issue were fMRI experiments by Gauthier et al. (1999) who compared the response to faces with that to face-like objects called Greebles. In human subjects the differences in fMRI images between up and down orientations of a human face had given a reproducible difference signal in the fusiform gyrus, leading to its assignment as a FFA The Greebles were designed to differ from each other, with individuals falling into classes based upon more prominent jaws and smaller nose-regions. Subjects were tested on their ability to recognize specific Greebles with differences between the ability of trained and untrained subjects to distinguish between the right-side up and upside down Greebles as a control. They measured fMRI difference signals from human subjects trained to recognize Greebles vs. signals from untrained controls to ascertain the degree of expertise and then measured the difference signal by subtracting the fMRI images from an upside down Greeble from one when it was right side up. They hypothesized that if an FFA-like activation arose with the development of expertise it would be evidence that the region is not innately specialized to recognize Greebles but rather is recruited through plasticity and experience.

In their original fMRI study (Gauthier et al., 1999) of untrained humans, no reproducible differences were observed in the FFA between the two orientations of the Greeble. However, after subjects had been trained to recognize Greebles, the different orientations of the Greebles gave fusiform gyrus activation quite similar to those raised by faces. Additional experiments to measure the difference signal from experts in identifying other familiar objects, e.g., animals, automobiles and planes also found activated regions in the FFA (Gauthier et al., 2000; Xu, 2005) The various objects, including Greebles, whose training history was definitely known, activated the same FFA region in experts as faces, leading to the conclusion that the response to facial recognition was not different from the response from experts who had been trained to recognize the objects. Therefore the postulation of an innate facial recognition region, different from the attributes of expertise, was not supported by the data which showed that the response to faces was similar to the response from experts trained to recognize familiar objects (Gauthier et al., 1999).

### Ultra High Resolution Studies of the FFA Provide Further Support of a Non-modular Interpretation

The findings of Gauthier et al. (1999) and others denying the modular nature of object recognition were criticized (Tsao et al., 2006) based on the resolution of the fMRI studies being 2 mm × 2 mm × 3 mm which, it was claimed, could lead to regions specialized for only facial recognition overlapping with other regions. To test this possibility studies were performed by at higher spatial resolution (approximately 1 mm^2^ in plane). The initial high resolution studies provided ambiguous results due to low signal to noise (Grill-Spector et al., 2006). However, a subsequent study at 7T, which provided higher sensitivity, showed conclusively that the same FFA region was activated by both facial recognition and by an expert’s recognition of familiar objects (McGugin et al., 2012). Hence, since the recognition of familiar objects like automobiles, birds and Greebles, where expertise was developed by training, were identical to the signals from faces, the simplest conclusion was that faces, along with these other familiar objects, led to the development of a region in the FG of increased brain activity via training, not by an innate FFA module existing for each of the familiar objects. A further argument against an innate FFA is that cognitive processes specializing in objects only in existence over the last two centuries such as automobiles and planes could not have been selected by evolution.

### Localized Neuroimaging Responses Do Not Imply Cognitive Processes: An Alternate Explanation of Imaging Evidence for Functional Segregation Based Upon Neuroplasticity and Image Averaging

Localized regions of activation in neuroimages, as found in the FG studies, are often cited as evidence for underlying separable cognitive processes. However, detailed examination of the FG studies, discussed above, where well localized imaging responses, albeit subject to the limitations described in Sections “Basic Structure of Standard Cognitive Models (SCM) and Their Application to Functional Neuroimaging” and “The Non-cognitive Behavioral Model (NBM)” of SCM experimental paradigms, are found when subjects have expertise in recognition, have been shown to not support the presence of an innate modular FFA. Instead localization and strength of the neuroimaging response depend on expertise and training. The question, however, remains regarding how do well localized regions of enhanced (or decreased) neuronal activity arise in an image.

Due to limitations in sensitivity for both PET and fMRI the images obtained during tasks, particularly cognitive tasks, must be repeated multiple times and added together. Often the results from several subjects are added. As such, a reproducible pattern of neuronal activity during a behavior will create a greater average neuroimaging signal than less reproducible patterns even if they are supported by similar overall amounts of neuronal activity. The NBM hypothesizes that a reproducibile pattern of brain activity in response to an input, results from repetition leading to the pattern being selected for using brain mechanisms for neuroplasticity.

### Conclusions

Neuroimaging studies of object and facial recognition show a dependence on expertise consistent with the NBM interpretation that neuroplasticity and experience play a key role in how the brain supports behavior. Expertise comes from recruitment of regions within the FG (and other brain regions) that depend upon training and experience as opposed to being an innate module. The ubiquity of facial recognition without any training presumably reflected the many exposures to faces at an early date thereby training people so that the fMRI data are thereby consistent with the expectations of the NBM. In Section “Application of the NBM to Study Cognitive Behaviors,” we discuss how reproducible neuronal activity (and the neuroimaging signals it is mapped by) develops through experience and why sensory processing regions show much higher reproducibility than cognitive regions.

We caution that the agreement with experiment depends upon selecting results that, while they seem valid to us, must be recognized as not uncontested. In addition all of the studies described above were performed using the SCM framework and were therefore designed to identify the location(s) of a previously hypothesized mental process, whether from an innate modular type structure or a similar structure derived from experience. Within the framework of this paper, which intends to describe the NBM model and its advantages in interpreting specific experimental results, we cannot claim that our presentation offers a balanced review of the many results available from relevant brain imaging experiments. However, by concentrating on experiments that have intended to distinguish between modular and non-modular interpretations we hope to have clarified the nature of the disagreement between SCM and NBM and to show that the NBM interpretation of the Greeble study as supporting reproducible training being the origin of FFA localized brain activity.

## Application of the NBM to Study Cognitive Behaviors

In this section, we further address the question of how NBM and other bottom up models can be used to study cognition and other complex behaviors. This question was highlighted soon after the discovery of fMRI in an interview with one of the authors (Shulman, 1996), and is largely based upon the lack of a direct theory of cognition (such as is embedded in the SCM) being tested (Morcum and Fletcher, 2007). We describe here a program for studying the brain/behavior relationship that uses neuroimaging to identify neuronal mechanisms supporting cognition based upon the similarity of patterns of brain activity and directly taking into account context and the experiences of the subjects.

### The NBM as an Explanation for the Lack of Unique Functional Segregation for Supporting Different Cognitive Processes

As described in Section “Basic Structure of Standard Cognitive Models (SCM) and Their Application to Functional Neuroimaging” neuroimaging studies using the SCM paradigm have not found clear functional segregation of cognitive processes which has been attributed to limitations in imaging reliability as well as non-linearities and regional interactions in the brain’s implementation of cognitive processes (Friston et al., 1996; Price and Friston, 2007; Gonzalez-Castillo et al., 2012). In the NBM, we propose that the lack of clear functional segregation is a consequence of the brain not being organized to support abstract cognitive processes as described by the SCM. Instead the specific neuronal instantiations of behavior depends largely upon training and experience acting upon mechanisms of neuroplasticity. Due to the large variations in the experiences of individuals patterns of neuronal activity supporting behaviors will also exhibit large variations. Furthermore differences in an individual’s history changes the context in which a behavior is performed or interpreted. For example, the meaning of concepts like patriotism, memory or beauty will vary considerably between individuals, with different histories, so that the brain activity induced by the same word will depend upon its context.

The higher reproducibility of the neuronal response to sensory stimuli is driven by the reproducible training in sensory tasks generated in everyday life, i.e., we all can accurately distinguish red from blue and up from down. The specific brain responses learned by early repetitive exposure to sensory stimuli show high functional segregation in that they become sensitized to a similar visual feature, e.g., moving lines or edges or red versus blue. The easy transferability of specific sensory phenomena relative to mental concepts is due to their being reproducibly defined by environmental input. Psychological concepts like working memory are not reproducible because they are not uniformly defined between individuals nor in the same individual in different contexts^2^.

### How Mental Processes Can Be Studied With the NBM

In NBM there is no equivalent to the traditional SCM interpretation of neuroimages in which the brain is functionally organized to support predefined mental processes. Instead similarities in the pattern of brain activity determines whether behaviors are related. For example suppose one wants, from an NBM guided experiment, to know the brain activities needed during a set of behaviors that can be described in somewhat general behavioral terms as working memory. In the NBM there is no definition of a concept of working memory that deploys the same mental process in different behaviors. The behaviors could be described as including something that could be called working memory only if you were willing to sacrifice accuracy to obtain such a generalization and were willing to overlook differences in the brain activity supporting the behaviors.

In the NBM paradigm, behaviors traditionally assigned to concepts such as memory would be studied using the same approach as the studies of consciousness and object/face recognition in Sections “Application of the NBM to Studies Determining Neural Correlates of Consciousness” and “fMRI Studies of Facial and Object Recognition.” In Section “fMRI Studies of Facial and Object Recognition,” it was determined from the images that the brain activity supporting expert object recognition and face recognition in the FG were similar, implying related neural mechanisms. Similarly, studies of “memory” would involve having the subject perform a series of behaviors that involve memory defined operationally as the ability to respond to questions about previous knowledge. However, any conclusion about whether the brain responses to the behaviors so defined as memory are similar would depend upon neuroimaging establishing that they use related neuronal mechanisms. The problem that might remain is whether the brain implementation has sufficient similarities between individuals to identify common mechanisms. It might be that this definition of “memory” might differ between individuals, given the same definition of behavior, like the synonyms in a dictionary that invariably accompany the definition of a term. In the SCM, mental processes often are produced by words that encompass their activity but it is unclear if the concepts embodied in words will be distinguished at the level of neural mechanisms. The breakdown of memory in SCM guided experiments into working memory and other kinds of memory suggests that different kinds of kinds of memory are distinguished and future work might be able to identify more useful distinctions while acknowledging their uniqueness as a consequence of the life experiences (external and internal) of the individual.

A potential limitation of the NBM approach is that the variation within and between subjects will be too high in order to find correlations, particularly given the limited signal to noise of fMRI. The large majority of studies have been performed using SCM paradigms in which there are significant constraints on the data analysis in order to achieve statistical significance, albeit at the cost of potentially forcing results to agree (see the section “Basic Structure of Standard Cognitive Models (SCM) and Their Application to Functional Neuroimaging” and references cited within). However, with improvements in fMRI sensitivity it has been possible to do relatively unconstrained analysis of the pattern of brain response to visual tasks and find reproducibility at least within subjects (Gonzalez-Castillo et al., 2012) as well as in resting state fMRI (Smith et al., 2013) networks that contain regions that have been assigned (albeit by an SCM approach) to cognition. Ultimately, however, experiments will need to be performed in order to answer the question of whether common mechanisms can be identified using an assumption free approach like the NBM.

### The NBM in the Study of Consciousness

The SCM goal was to find a brain region activated during the cognitive acts defined as consciousness. This goal has failed, because of the SCM requirements first to find agreement on the definition of “consciousness” and second to identify the brain regions activated. These failings have been avoided by planning and interpreting the search by NBM. In this approach the behavior is identified by observing when the subject in the state of consciousness while the brain region to be associated with that behavior need not be localized. We see here two advantages of the NBM method. To study consciousness by an SCM type experiment means locating it in a brain region, which also requires that it is necessary to agree about the definition of the term. Since neuroscience has been severely criticized by philosophers and others for not being able to define consciousness (Nagel, 2016) efforts by studies using the SCM to study consciousness would only succeed if first there had been such a consensus and second if it resulted in well defined functional segregation. By contrast using the NBM experimental approach of direct statistical correlation it was found that the strongest predictor of consciousness was the total, global, energy consumption by the brain when the person was identified by behavior as being in the state of consciousness.

### Conclusion

In conclusion, we propose that the brain’s support of cognitive behavior is highly experience-dependent and sensitive to the context of the study. As people have normally been trained during their early years to recognize sensory stimuli their brains will show similar patterns of neuronal activity when the same stimuli are presented, and when summed will give reproducible and well localized neuroimaging activity maps. Furthermore once trained by such reproducible exposures the brain activity will continue to be activated by the stimulus. However, as generalized behaviors like remembering, calculating or paying attention depend on their context and on the person’s history, the neuronal activity needed to support them will vary and generally will not yield highly reproducible functional neuroimages. Using the neuroimaging activity maps as a guide, it is proposed by NBM to identify behaviors that are supported by related brain mechanisms. However, it is unlikely, based on present results, that the degree of reproducibility required to support models will be found in cognitive modules and therefore it is necessary to look for empirical mechanisms to tighten the correlation between brain activity and behavior.

## Epistemological Basis of the NBM

To the degree that NBM can find reliable relations between observable behavior and brain patterns of neuronal activity, we have fulfilled a goal of our modified behaviorism. Therefore, we stop to ask what have we achieved? Does NBM have any generalized importance beyond the knowledge that a certain brain region responds to a certain person’s behavior? Does it tell us anything of general usefulness or is it limited to the particular experimental conditions from which we drew correlations? This is the recurring question faced by all models of brain function that are not based on conceptualizations of mental processes attributed to behavior such as are offered by the SCM.

One of the most significant contributions to this problem was offered by Charles Sanders Peirce in his extension of philosophical pragmatism (Peirce, 1958). Peirce suggested that a unifying evaluation of such differing results could be found by considering the consequences of these separate but similar relationships upon human actions. In his method for identifying the meaning of a pragmatist conclusion, Peirce proposed a criterion for understanding the consequences of assigning a generalized conceptualization to a well-defined behavior, which we have extended to its correlation with a brain activation. For Peirce the value of concepts behind behaviors like working memory, free will or unselfishness was not to be determined by how accurately they described the phenomenon, but rather by the meaning they had for human affairs. His definition was intended to return the term to scientific purview by defining it in terms of scientific “thought” experiments as follows- “Consider, what effects, that might conceivably have practical bearings, we conceive the object of our conception to have. Then, our conception of these effects is the whole of our conception of the object” (Ibid, p. 192). This includes the likelihood that a brain response for a somewhat generalized act will include different brain responses reflecting the usage allowed by the several synonyms of the term in a dictionary. Since the meaning of a concept depends on the effects it may have on humans, this definition has not assigned a value to this meaning but has identified its usage in a common expression.

As discussed by Craver (2007), we need some principled and empirical way of saying when observable behaviors can be correlated without previous assumptions of similarity. It is here that Peirce’s turning to the possible effects of the understanding of the behavior upon human actions, as the criterion of meaning, rescues us from the criticism of having found only a subjective result. In decomposing the behavior into little behavioral steps, not into cognitive concepts, we might find linked brain correlates of the jump, in brain neuronal activity. Furthermore unlike the SCM there is not a starting assumption about mental processes the brain is performing, instead there are details of behavior that can be teased out which are ignored by assuming classes of behaviors are similar. Furthermore by relating detailed behaviors to the effects upon human activity following Peirce or upon the correlated brain activity as proposed in NBM we would be on the way to coordinating brain activations during a behavior, a fundamental goal of neuroscience.

Although our proposal of an empirically based model of brain function has not, to our knowledge, been previously generalized from neuroimaging data, still specific, similar empirical models have been proposed (Koch, 2017) by authors who previously had leaned toward interpreting consciousness by modular-like concepts (Koch, 2004). This move, linking conscious behavior to a measurement of its electrical activity formally resembles our NBM model rather than the many former studies of consciousness that searched for a brain activity linked to a cognitive concept. Tononi et al. (2016) propose that measuring brain electrical activity after stimulating the brain with magnetic pulses provides a reliable measurement of consciousness. They proposed that an empirical level of brain activity, which they obtained from comparison of conscious and unconscious subjects, could be used to define consciousness.

In substituting correlations for causal relationships between observed behavior and brain activities, Koch and Tononi’s model, similar to our NBM and to Peirce’s proposal for future experiments resembles the recent progress in machine learning to find relationships between data and behavior without prior hypotheses. The vast amount of data obtained even in a single functional neuroimaging study in principle would be well suited to this type of analysis. In addition this approach could be integrated with models of brain function derived from bottom up lower level models of brain circuitry and general informational principles. Significant attempts have been made in these areas that potentially could be integrated into modeling neuroimaging studies (e.g., Bedau, 1997; van der Maas et al., 2006; Kitzbichler et al., 2009; Hellyer et al., 2017). By taking advantage of the developing methods of machine learning our empirical approach to neuroimaging provides an exciting future for understanding brain functions.

## Author Contributions

RGS and DLR contributed to the writing of the manuscript, the hypotheses and theories presented, and the analysis of data reviewed.

## Conflict of Interest Statement

The authors declare that the research was conducted in the absence of any commercial or financial relationships that could be construed as a potential conflict of interest. The reviewer AG declared a past co-authorship with the authors to the handling Editor.

## Footnotes

1 It has been shown by comparison to electrical recording and ^13^C MRS measurements of glutamate and GABA release and recycling that over a wide interval neuronal activity is proportional to the incremental energy that the brain needs above non-signaling processes (Sibson et al., 1998; Hyder et al., 2013a,b). Therefore methods such as FDG-PET and calibrated fMRI have the potential of providing quantitative maps of neuronal signaling (e.g., Stender et al., 2015, 2016). In order to not obscure the relationships between brain activity and behavior due to issues regarding the veracity with which neuronal signaling is tracked by imaging we assume in this paper (except where stated otherwise) that imaging is providing whole brain maps of neuronal signaling (or the difference in signaling between two states as with fMRI).

2 In contrast to the human studies, studies of non-human primates performing cognitive tasks have been shown to give highly reproducible localized patterns of activity after they were strongly trained to perform such tasks. These observations (e.g., Goldman-Rakic, 1995) have encouraged neuroscientists to believe that cognitive concepts like WM are represented in the brain in a neuroanatomically reproducibile and well defined manner, despite functional neuroimaging studies in humans showing poor localization (see the section “Basic Structure of Standard Cognitive Models (SCM) and Their Application to Functional Neuroimaging” and references therein). Our alternate interpretation is that observation of the expected brain activities in non-human primates after such intense training, but not in un-trained human subjects, argues against intrinsic functional segregation of brain activity supporting the cognitive process of working memory. Instead the functional segregation derives from repeated training and eventual selection of a fixed neuronal pathway for supporting the behavior.
